# Primary Pulmonary Mucoepidermoid Carcinoma: Histopathological and Moleculargenetic Studies of 26 Cases

**DOI:** 10.1371/journal.pone.0143169

**Published:** 2015-11-17

**Authors:** Zhen Huo, Huanwen Wu, Ji Li, Shanqing Li, Shafei Wu, Yuanyuan Liu, Yufeng Luo, Jinling Cao, Xuan Zeng, Zhiyong Liang

**Affiliations:** 1 Department of Pathology, Peking Union Medical College Hospital, Chinese Academy of Medical Sciences & Peking Union Medical College, Beijing 100730, China; 2 Department of Thoracic Surgery, Peking Union Medical College Hospital, Chinese Academy of Medical Sciences & Peking Union Medical College, Beijing 100730, China; Cincinnati Children's Hospital Medical Center, UNITED STATES

## Abstract

**Introduction:**

Pulmonary mucoepidermoid carcinoma (PMEC) is an uncommon neoplasm of the lung and the main salivary gland-type lung carcinoma. The aims of this study were to review the clinicopathological and immunohistochemical features of PMEC and characterize the genetic events in PMEC.

**Methods:**

We reviewed the pathology cases in our hospital and found 34 initially diagnosed PMEC cases, 26 of which were confirmed as PMEC after excluding 8 cases of MEC-like pulmonary carcinoma. The clinicopathological characteristics of the 26 PMEC cases and the 8 cases of MEC-like pulmonary carcinoma were retrospectively reviewed. MAML2 rearrangement was detected by fluorescence In Situ Hybridization (FISH). Immunostains of ALK, calponin, collagen IV, CK7, EGFR, HER2, Ki-67, Muc5Ac, p63, p40, and TTF-1 were performed. DNA was extracted from 23 cases of PMEC. Mutation profiling of the EGFR, KRAS, BRAF, ALK, PIK3CA, PDGFRA, and DDR2 genes were carried out using next-generation sequencing (NGS), Sanger sequencing, and quantitative polymerase chain reaction (QPCR) in 9 successfully amplified cases.

**Results:**

Twenty-six cases of PMEC (18 low-grade, 8 high-grade) included 13 men and 13 women aged 12–79 years. Twenty-two cases had a central/endobronchial growth pattern, and 4 cases had a peribronchial growth pattern. Immunohistochemically, CK7, Muc5Ac, p40, and p63 were positive in all cases (26/26);EGFR was positive in 11 cases (11/26); TTF-1, Calponin, HER2 and ALK were negative in all cases (0/26). MAML2 rearrangement was identified in 12 of 18 PMEC cases. No mutations were detected in any of the 7 genes in the 9 cases that qualified for mutation analysis. Twenty-three PMEC patients had follow-up information with a median interval of 32.6 months. Both the 5- and 10-year overall survival rates (OS) were 72.1%, and a high-grade tumor was an adverse prognostic factor in PMEC. There were 8 cases of MEC-like pulmonary carcinoma aged 36–78 years: 2 cases were located in the bronchus, and 6 cases were located in the lung. p63 and TTF-1 were positive in all cases (8/8), p40 was positive in 5 cases (5/8), and ALK was positive in 5 cases (5/8). No cases of MAML2 rearrangement were detected, but there were 5 cases of ALK rearrangement.

**Conclusions:**

PMEC is a primary malignant pulmonary tumor with a relatively good prognosis that is historically characterized by the presence of mucous cells and a lack of keratinization. There are distinct differences between PMEC and MEC-like pulmonary carcinoma in tumor location preference, immunophenotype, and molecular genetics, and the differential diagnosis is critical due to the therapeutic and prognostic considerations.

## Introduction

Primary pulmonary mucoepidermoid carcinoma (PMEC) is a rare neoplasm that accounts for <1% of all lung carcinomas. It is presumed to originate from the minor salivary glands lining the tracheobronchial tree and is the main salivary gland-type carcinoma of the lung [1]. Recently, important genetic advances, including chromosomal translocations t (11; 19) (q21; p13) and t (11; 15) (q21; q26), have been made in the understanding of the molecular pathogenesis of mucoepidermoid carcinoma (MEC). These translocations produce a CRTC1/3 (cAMP-response element binding protein-regulated transcriptional co-activator 1/3)-MAML2 (mastermind-like protein 2) fusion gene [2–12]. The CRTC1-MAML2 and CRTC3-MAML2 fusion transcripts are present in approximately 30–80% and 6% cases of MEC, respectively [2–4, 6]. Some recent studies have demonstrated that the fusion is a clinically useful prognostic biomarker for MEC, and the highest incidence of the CRTC1-MAML2 fusion is found in low- and intermediate-grade MEC with favorable prognosis [7–9]. However, some subsequent studies showed that the fusion may occur infrequently in high-grade MEC with a dismal prognosis [10, 11]. To date, the MAML2 rearrangement in PMEC has been reported in fewer than 5 studies. It was found in 50%-100% of PMEC cases and in 12.5–43% of high-grade PMEC cases. The relationship of the MAML2 rearrangement and the prognosis in PMEC is not clear at present because of too few case studies [12–15].

Although many molecular genetic studies indicate that there are some genetic mutations in non-small cell lung cancer (NSCLC), including EGFR, KRAS, PIK3CA, BRAF, ALK, DDR2, and PDGFRA [16, 17], only a few studies have focused on the genetic events of salivary gland-type lung carcinomas. A few studies have reported that the genetic mutations in salivary gland malignant tumors include EGFR, KIT, BRAF, HRAS, PIK3CA, and HER2 [6, 18, 19]. Gene alterations in HER2, EGFR, and KRAS have been reported in PMEC [20–26].

In the current study, we reviewed a retrospective series of 26 patients with primary PMEC in our hospital from 2000 to 2014. We emphasized their clinical and pathologic features, treatments, and the possible prognostic factors, focusing especially on the MAML2 rearrangement and its relationship to prognosis. We also evaluated the EGFR, KRAS, BRAF, ALK, PIK3CA, PDGFRA, and DDR2 gene status in PMEC using three different methods, including next-generation sequencing (NGS), Sanger sequencing, and quantitative polymerase chain reaction (QPCR).

## Material and Methods

### Patients and Specimens

We reviewed all surgical lung biopsy or resection records in Peking Union Medical College Hospital from January 1, 2000, to December 31, 2014, and identified a total of 26 cases of PMEC, accounting for 0.25% of all the 10,500 primary malignant pulmonary tumors. In addition to these 26 patients, we also found 8 patients who had been misdiagnosed with PMEC who instead had MEC-like components based on a combination with histology, immunohistochemistry, and fluorescence in situ hybridization (FISH) results. These 8 patients were excluded from the present analysis for primary PMEC, and we analyzed them separately as an important differential diagnosis, MEC-like pulmonary carcinoma. The patients’ medical records were collected and reviewed, and no patient had a history of salivary gland tumor.

All samples were fixed in 10% neutral buffered formalin, routinely processed, and embedded in paraffin. Hematoxylin-eosin stained sections were observed using optical microscopy and reviewed independently by three experienced pathologists. They classified the tumors as either low- or high-grade based on the World Health Organization (WHO) criteria for PMEC [1]. The investigated parameters included anatomical location, histological type, neural and/or vascular involvement, lymph node metastasis, mitotic count, and necrosis status. We gathered follow-up data from outpatient follow-ups. Ethics committee of Peking Union Medical Collage Hospital specifically approved this study and patients provided their written informed consent to participate in this study.

### Immunohistochemical staining and Scoring

Immunostains of ALK, calponin, collagen IV, CK7, EGFR, HER2, Ki-67, Muc5Ac, p63, p40 (ΔNp63), and TTF-1 were performed in all 26 cases of PMEC and 8 cases of MEC-like carcinoma (Table 1) according to the manufacturer's instructions. They were performed on 4-μm-thick unstained sections cut from representative formalin-fixed paraffin-embedded (FFPE) blocks. For all markers, positive controls and negative controls were used. For calponin, CK7 and Muc5Ac, signals appearing as tan particles in the cytoplasm were considered positive. For p63, p40, TTF-1, and Ki-67, tan particles in the nucleus were considered positive. Ki-67 labeling index was determined by estimating Ki-67 immunostaining in the hightest proliferation areas(hot spots), and the percentage of tumor cells with nuclear immunostaining was claculated by counting of 1000 tumor cells at high-power view fields (HPFs) (10 HPFs, 100 cells per HPF). For Her2 and the EGFR protein, uniform intense reactivity or non-uniform or weak reactivity with obvious circumferential distribution in >10% of cells in the membrane was considered positive. An immunostain of ALK was performed on a benchmark Ultra Immunostainer (Ventana, USA). Distinct cytoplasmic staining with at least moderate intensity in any proportion of the tumor cells was considered positive.

**Table 1:** List of various antibody markers in the present study.

Antibody markers	Clone	Dilution	Producer
ALK	D5F3	Prediluted	Ventana(Roche), USA
calponin	EP63	Prediluted	ZSGB-BIO, China
CK7	EP16	Prediluted	ZSGB-BIO, China
Collagen IV	CIV22	1:100	Dako, Glostrup, Denmark
EGFR	EP11	Prediluted	ZSGB-BIO, China
HER2	EP3	Prediluted	ZSGB-BIO, China
Ki-67	MIB-1	1:100	Dako, Glostrup, Denmark
Muc5ac	MRQ19	Prediluted	ZSGB-BIO, China
p63	UMAB4	Prediluted	ZSGB-BIO, China
p40 (ΔNp63)	BC28	Prediluted	ZSGB-BIO, China
TTF-1	SPT24	Prediluted	Fuzhoumaixin, China

### FISH

FISH was performed on FFPE sections of 26 cases of PMEC and 8 MEC-like tumors using a commercially available MAML2 Dual Color Break Apart Probe (Z-2014-200, Zytovision, Germany) following the manufacturer's instructions. Cells with two fusion signals, one orange and one green fluorochrome, were scored as normal. Cells with a rearrangement in the MAML2 gene had one normal fusion signal and one orange and one green signal at a distance from each other. The distance between the two separated signals was estimated using twice the size of the biggest signal size. Positive cases were defined as more than 15% break-apart signals in 50 tumor cells. A total of 100 tumor cells were counted, and the percent split signal was recorded. Salivary gland mucoepidermoid carcinoma tissue was used as a positive control, and normal parotid gland tissue was used as a negative control.

The same FISH method was performed on FFPE sections of ALK-positive cases using a commercially available ALK Dual Color Break Apart Probe (Vysis LSI, Abbott Molecular, USA). FISH-positive cases were definedas having two positive ALK rearrangement patterns. One was the breakapartpattern with one fusion signal and two separated orange andgreen signals. Anotherdefinition was an isolated red signal pattern with one fusion signaland one red signal without a corresponding green signal. ALK-positive lung adenocarcinoma was used as a positive control, and normal lung tissue was used as a negative control.

### DNA extraction

Genomic DNA from 23 cases of PMEC was extracted from freshly cut FFPE tissue sections using a QIAamp DNA Mini Kit (Qiagen, Germany) according to the manufacturer’s instructions. The tumor area was identified through hematoxylin-eosin staining, and tissue from this area on unstained sections was scraped for DNA extraction. The extracted DNA was then quantified using the Qubit dsDNA BR assay (Life Technologies, USA). Out of 23 cases, 9 cases of PMEC were successfully amplified. Mutational analysis was carried out using three different methods, including next-generation sequencing (NGS), Sanger sequencing, and quantitative polymerase real-time chain reaction (QPCR).

### NGS and data processing

Targeted NGS was performed, with 10 ng of DNA as the template to generate the amplicon library for sequencing. Libraries were prepared using Ion AmpliSeq Library Kit 2.0 (Life Technologies, USA) and the Lung Cancer Mutation Panel (ACCB Biotech, China), which is designed to detect mutations within 16 exons of 7 lung cancer driver genes(EGFR, KRAS, BRAF, ALK, PIK3CA, PDGFRA, and DDR2 genes) (Table 2). Adapter ligation, nick repair, and PCR amplification were performed according to the manufacturer’s protocol. Libraries were then quantified using a Qubit dsDNA HS Assay Kit and a Qubit 2.0 fluorometer (Life Technologies, USA), with samples diluted to a concentration of 3 ng/mL and pooled in equal volumes. Emulsion PCR and enrichment steps were carried out using an Ion OneTouch Template Kit on the Ion OneTouch system (Life Technologies, USA) according to the manufacturer’s protocol. Following enrichment, the amplicon libraries were subjected to sequencing on the Ion Torrent PGM system (Life Technologies, USA) using 318 chips and barcoding with an Ion Xpress Barcode Adapters 1–16 Kit (Life Technologies, USA). After sequencing, reads were mapped to the reference genome (hg19) using the Torrent Mapping Alignment Program (TMAP). Variants were identified using Torrent Variant Caller (versions 3.6.6; Life Technologies, USA). The Integrative Genomics Viewer (Broad Institute, USA) was used to visualize variants against the reference genome to confirm the accuracy of the variant calls by checking for possible strand biases and sequencing errors.

**Table 2:** List of 16 exons of 7 genes in the present study.

Genes	Exons
EGFR	Exons 18, 19, 20 and 21
KRAS	Exons2 and 3
BRAF	Exons 11 and 15
PIK3CA	Exons 9 and 20
ALK	Exons 23 and 25
DDR2	Exon 18
PDGFRA	Exons 12, 14 and 18

### Sanger sequencing

Mutations within 16 exons of the 7 lung cancer driver genes were also screened by PCR-based 2-bidirectional direct Sanger sequencing using primers. The sequencing results were interpreted using Chromas software version 1.45 (Technelysium Pty, Australia).

### QPCR

The Human Mutation Qualitative Detection Kit (ACCB Biotech, China) was used according to the manufacturer’s instructions. Real-time PCR was run on a Rotor-Gene QPCR Platform (Qiagen, Germany). The cycling conditions for quality control (QC) runs and for mutation assays were as follows: 10 min incubation at 95°C, followed by 40 cycles of 95°C for 15 s, and 60°C for 1 min. Fluorescence was measured at 60°C. Data regarding each mutation were interpreted according to the kit manual after curve analysis and calculation of ΔCt values.

### Statistical Analysis

The 23 patients with follow-up data were further subjected to survival analysis. Survival curves were calculated according to the Kaplan-Meier method and compared using the log-rank test. The MAML2 rearrangement and the tumor location comparisons were conducted using Fisher’s exact chi-square test. The level of significance was defined as P ≤0.05 (two tailed). Patient median follow-up time was calculated using a reverse Kaplan-Meier analysis. All statistical analyses were performed using SPSS software for windows, version 22 (SPSS Inc., USA).

## Results

### 1. Clinical data

The 26 cases of PMEC (Table 3 and S1 Table), with a mean age of 46.5 years, included 13 men and 13 women. Twenty-two patients had symptoms, with the most common being cough, hemoptysis, and dyspnea. According to the results of bronchoscopy and/or a chest CT scan, 22 tumors were located in the trachea or bronchus, whereas the remaining 4 tumors were located in the lung, which did not have a clear relationship to the bronchus. Preoperative pulmonary ventilation function was performed in 20 patients using spirometry, and 3 patients (15%) exhibited dysfunction. The tumor grew into the lumen of the trachea or bronchus in all 16 patients undergoing bronchoscopy, 10 of which showed complete luminal obstruction. Chest CT scans were performed in 25 cases, and in 4 cases, the tumor was located in the peripheral lung. The diameters of the lesions on chest CT scan ranged from 0.7 cm to 6.0 cm (mean, 2.5 cm).

**Table 3:** Clinical and follow-up data of 26 patients with pulmonary mucoepidermoid carcinoma.

	Number (%)		Number (%)
**Age, yr**		*Lobe and segmental bronchus*	*16(72*.*7)*
Mean	**46.5**	Upper right	2(12.5)
Range	**12–79**	Middle right	3(18.75)
**Gender**		Lower right	2(12.5)
Female	**13(50)**	Upper left	3(18.75)
Male	**13(50)**	Lower left	6(37.5)
**Smoking**		***Within lung***	**4(15.4)**
Never	**19(73.1)**	Upper right	1(25)
Have smoked	**7(26.9)**	Lower right	2(50)
**Symptoms**		Lower left	1(25)
***Present***	**22(84.6)**	**Operation method**	
Cough	15(68.2)	***Resection***	**23(88.5)**
Hemoptysis	11(50)	Lobectomy	17(73.9)
Dyspnea	7(30.4)	Wedge resection	1(4.3)
Chest pain	2(9.1)	Partial tumor resection	2(8.7)
Fever	1(4.5)	Tracheal or bronchial segmental resection	3(13.1)
Hoarseness	1(4.5)	***Biopsy***	**3(11.5)**
***Absent***	**4(15.4)**	**Radiotherapy and/or Chemotherapy(n = 23)**	
**Duration symptom, mo**		***Received***	**9(39.1)**
Median	**5.5**	Radiotherapy	4(44.5)
Range	**0.5–20**	Chemotherapy	3(33.3)
**Pulmonary function testing (n = 20)**		Both	2(22.2)
Restrictive	**1(5)**	***None***	**14(60.9)**
Obstructive	**2(10)**	**Follow-up time. mo(n = 23)**	
Mixed	**0**	Mean	**32.6**
Normal	**17(85)**	Range	**7–170**
**Bronchoscopy (n = 16)**		**Prognosis (n = 23)**	
Neoplasm in lumen	**16(100)**	***Survival***	**19(82.6)**
Normal	**0**	Disease free	18(94.7)
**Location (n = 23)**		Survival with tumor	1(5.3)
***Trachea or bronchus***	**22(84.6)**	***Death***	**4(17.3)**
*Trachea*	*4(18*.*2)*	**Metastasis (n = 3)**	
*Main bronchus*	*2(9*.*1)*	Brain	1
Left	1(50)	Bone	2
Right	1(50)	Adrenal gland	1

Eight cases of MEC-like pulmonary carcinoma, initially diagnosed as PMEC, with a mean age of 58 years, included 4 men and 4 women. Two cases had tumors located in the bronchus. The remaining 6 cases had tumors located in the lung diagnosed by bronchoscopy and/or by chest CT scan. MEC-like pulmonary carcinoma showed a location preference in the lung compared with PMEC, and the difference was statistically significant (P = 0.03). The diameters of the lesions on chest CT scan ranged from 1 to 4.0 cm (mean, 2.8 cm). The clinical data of the 8 cases of MEC-like pulmonary carcinoma are listed in Table 4.

**Table 4:** Clinicopathological, immunohistochemical, and fluorescence in situ hybridization data of 8 patients with mucoepidermoid carcinoma-like pulmonary carcinoma.

No.	1	2	3	4	5	6	7	8
Age, yr	48	54	46	36	66	71	78	65
gender	Female	Female	Male	Male	Female	Male	Fmale	Male
Location	Upper right lobe	Lower left lobe	Lower left lobe	Upper right lobe bronchus	Upper rightlobe	Lower left lobe	Upper left lobe bronchus	Middle right lobe
Symptoms	No	No	Cough and hemoptysis	Cough, hemoptysis and chest pain	Cough, fever and back pain	No	Cough	Cough and fever
Surgery	Yes	Yes	Yes	Yes	Yes	Yes	No	Yes
Final diagnosis	Adenocarcinoma	Adenocarcinoma	Adenocarcinoma	Adenosquamous carcinoma	Adenosquamous carcinoma	Adenocarcinoma	Adenocarcinoma	Adenocarcinoma
IHC findings										
TTF-1	**+**	**+**	**+**	**+**	**+**	**+**	**+**	**+**
CK7	**+**	**+**	**+**	**+**	**+**	**+**	**+**	**+**
uc5Ac	**+**	**+**	**+**	**+**	**+**	**+**	**+**	**+**
p63	**+**	**+**	**+**	**+**	**+**	**+**	**+**	**+**
p40	**-**	**+** ^#^	**-**	**+**	**+**	**+**	**+** ^#^	**-**
EGFR	**+**	**+**	**+**	**+**	**+**	**+**	**+**	**+**
ALK	**+**	**+**	**+**	**+**	**-**	**-**	**-**	**+**
HER-2	**-**	**-**	**-**	**-**	**-**	**-**	**-**	**-**
Ki-67	8%	10%	15%	3%	5%	10%	3%	5%
ALK arrangement	Yes	Yes	Yes	Yes	/	/	/	Yes
MAML2 arrangement	No	No	No	No	/	No	/	No
Follow-up time, mo	9	23	5	7	4	51	/	3
Recurrence or metastasis	Yes	No	No	Yes	No	No	/	No
Death	No	No	No	No	No	No	/	No

^#^ Scanty

### 2. Pathologic findings

**Fig 1:**
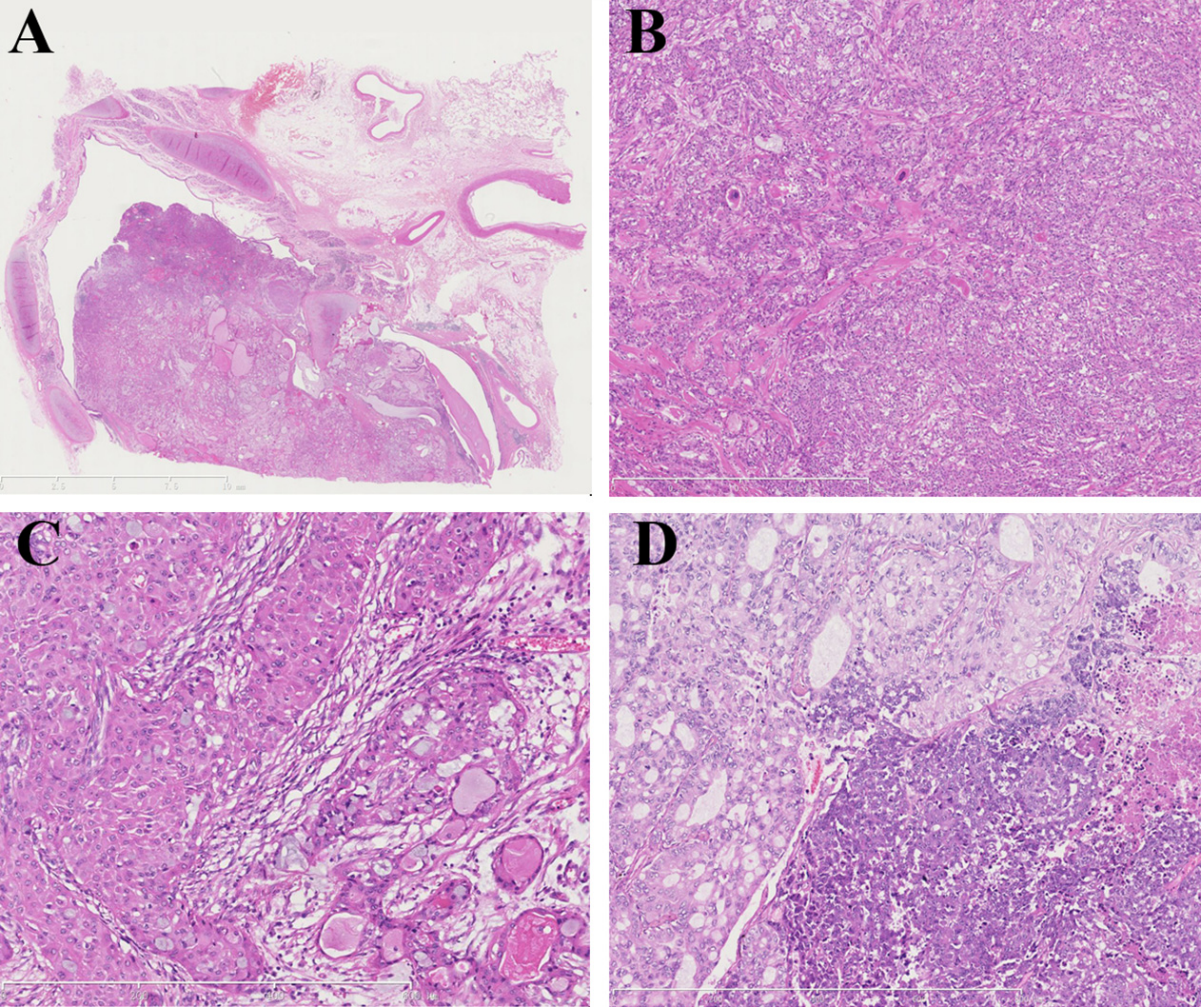
Microscopic images of primary pulmonary mucoepidermoid carcinoma. (A) Cross-section of the lobe bronchus demonstrating primary pulmonary mucoepidermoid carcinoma with an endobronchial growth pattern (H&E, lower power). (B) The same case with solid nests and a cystic component that comprises the tumor with hyalinization stroma, foci of calcification, and mucus in the cystic component (H&E, x75). (C) The tumor was composed of mucous, intermediate, and epidermoid cells without keratinization (H&E, x150). (D) Another primary pulmonary mucoepidermoid carcinoma case showing dedifferentiation with severe nuclear atypia, necrosis, and salient mitotic figures (right side) and the upper left corner showing the typical mucoepidermoid carcinoma area (H&E, x150).

Except for 3 patients who received a bronchial biopsy only, the remaining 23 patients received surgical resection, 19 cases of which had a central/endobronchial growth pattern (Fig 1A) and 4 had a peribronchial growth pattern. All tumors had a single nodule. Microscopically, 18 cases were low grade, and 8 cases were high grade with marked cellular atypia and mitotic figures in more than 4/10 HPFs. All tumors were composed of mucous, intermediate, and epidermoid cells without keratinization (Fig 1B and 1C). The tumor stroma showed different degrees of hyaline degeneration in all cases and had an amyloid-like appearance in 4 cases. Calcification was found in 11 PMEC cases. Only in high-grade tumors with dedifferentiation did the mitotic figure exceed 10/ 10HPFs (Fig 1D). Necrosis was observed in five high-grade tumors. Perineural invasion was found in only one patient. Lymph node metastasis was found in one patient (Table 5).

**Table 5:** Pathological features of 26 patients with pulmonary mucoepidermoid carcinoma.

	Number(%)		Number(%)
**Grade**		**Lymphatic metastasis (n = 22)**	
Low Grade	18	Present	1
High Grade	8	Absent	21
**Location (n = 23)**		**Blood vessel invasion**	
Central/endobronchial	19	Present	0
Peribronchial	4	Absent	26
**Mitotic figures**		**Resection margin (n = 22)**	
≥4/10HPFs	8	Positive	0
<4/10HPFs	18	Negative	22
**Cellular atypia**		**Calcification**	
Mild	18	Present	11
Moderate or Severe	8	Absent	15
**Necrosis**		**Hyaline degeneration**	
Present	5	Present	26
Absent	21	Absent	0
**Perineuralinvasion**		**Pleural involvement (n = 22)**	
Present	1	Present	1
Absent	25	Absent	21

**Fig 2:**
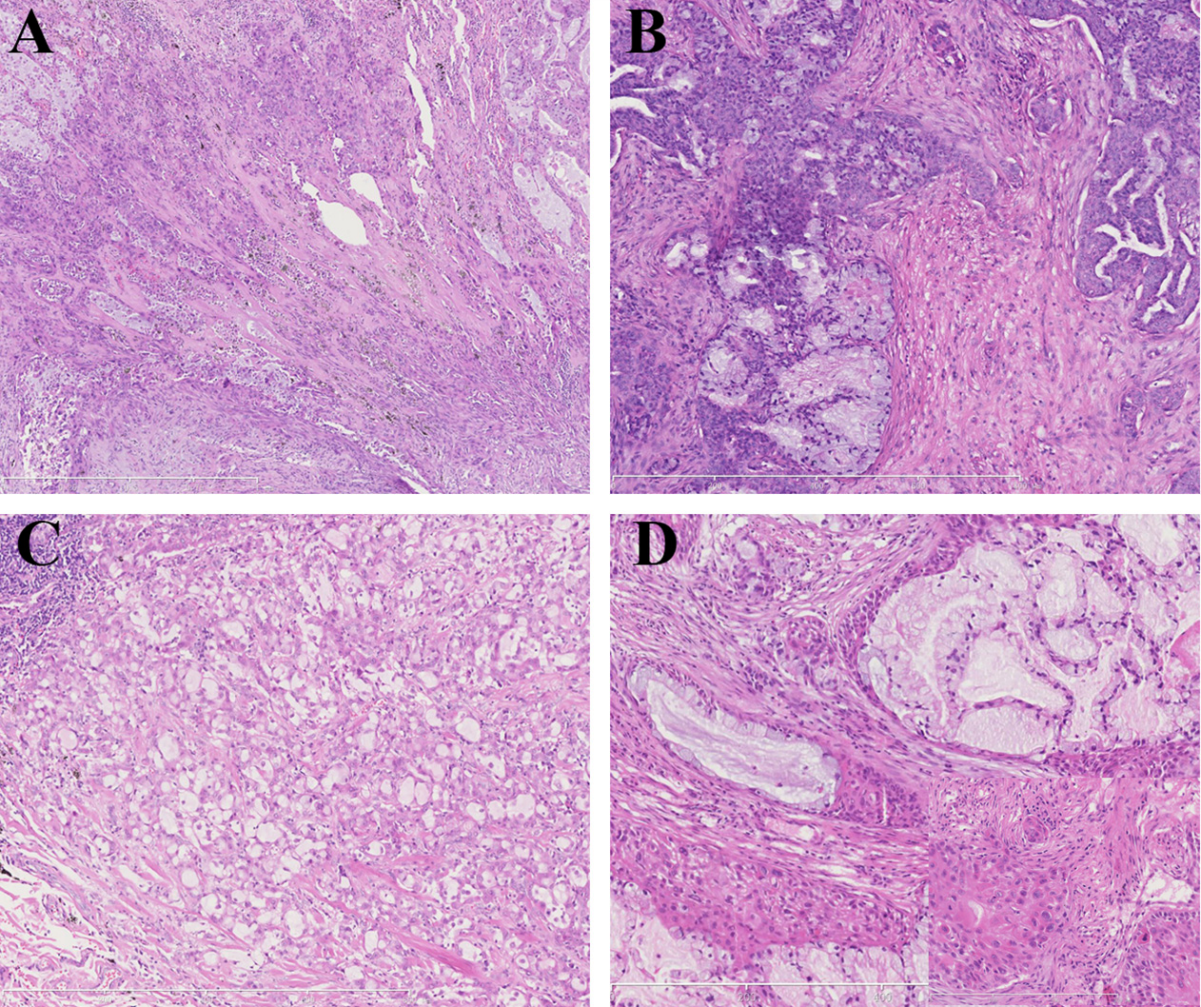
Microscopic images of MEC-like pulmonary carcinoma. (A) Case 1 showing solid nests in the left upper corner and mucin-filled cysts and mucous cells on the right side (H&E, x75). (B) Case 2 showing many mucous cells in the solid nests (H&E, x150). (C) Case 3 showing a cribriform-like structure with mucous cells (H&E, x150). (D) Case 4 was a mucoepidermoid carcinoma-like adenosquamous carcinoma (H&E, x150) (the illustration in the lower right corner clearly shows keratinization).

All 8 cases of MEC-like pulmonary carcinoma had clear mucous cells and solid nests; 6 cases were re-diagnosed as adenocarcinomas with mucin-filled cystic or mucin-filled cells in solid nests and other glandular structures or cribriform architecture (Fig 2A–2C). The other two cases displayed definite keratinization and was re-diagnosed as adenosquamous carcinoma with a mucin-filled cystic structure (Fig 2D).

### 3. Immunohistochemical findings

**Fig 3:**
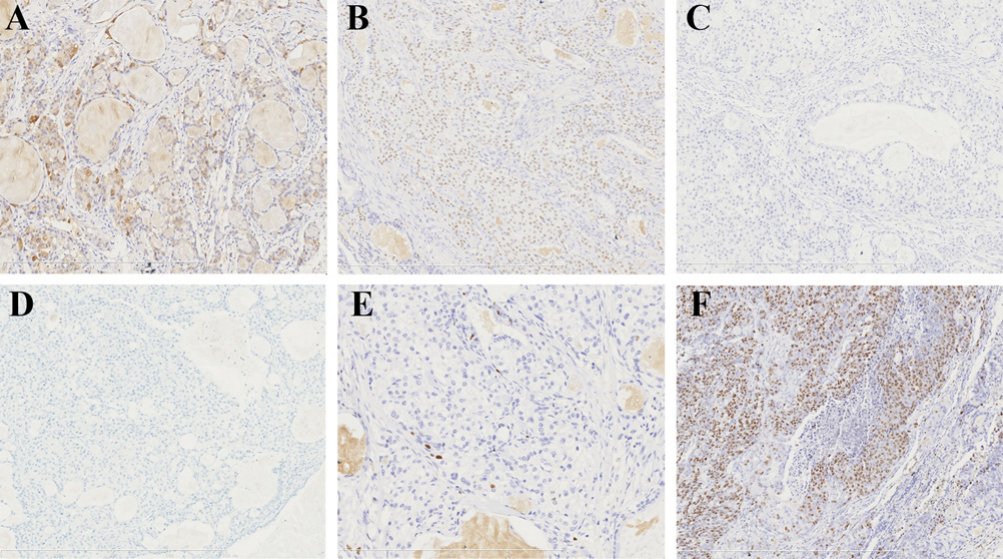
Immunostains of PMEC. (A) Muc5AC highlighted the partial mucous cells within the tumors (x150). (B) p63 was positive in both intermediate and epidermoid cells (x150). (C) TTF-1 was negative in all three cells (x150). (D) ALK was negative (x150). (E) Ki-67 was positive in the nucleus in a low-grade tumor, and the labeling index is 2% (x150). (F) Ki-67 was positive in the nucleus in the dedifferentiation area, and the labeling index is about80% (x150).

CK7, Muc5AC (Fig 3A), p63 (Fig 3B), and p40 were positive in all 26 PMEC cases (26/26); EGFR was positive in 11 cases (11/26); TTF-1 (Fig 3C), ALK (Fig 3D), and HER-2 were negative in all cases (0/26). The Ki-67 (Fig 3E and 3F) labeling index ranged from 2% to 80% (mean 9.7%). The mean index in low-grade and high-grade tumors was 4.1% and 22.4%, respectively. (Details in S1 Table).

**Fig 4:**
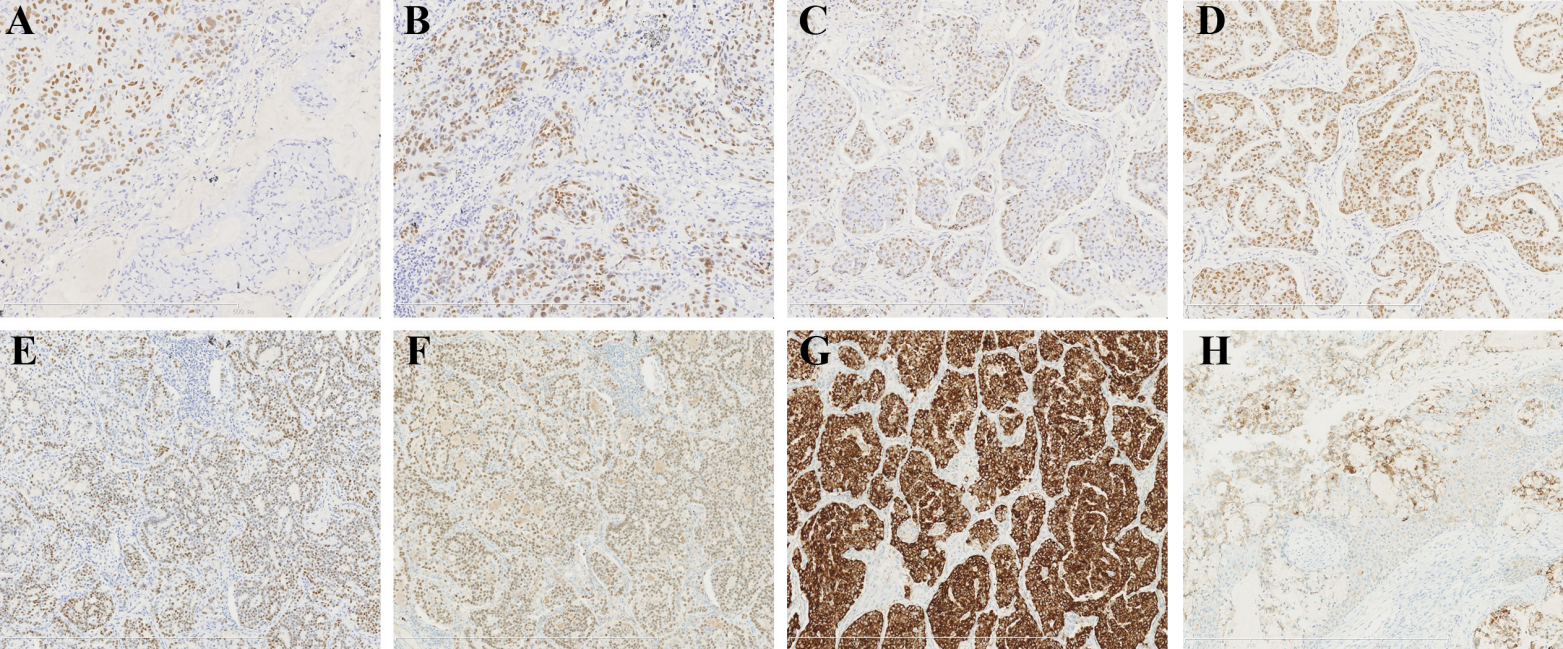
Immunostains of MEC-like pulmonary carcinoma. (A) P63 was positive in some cells in the same case in 2A (x150). (B) TTF-1 was positive in some cells in the same case in 2A (x150). (C) p63 was positive in some cells in the same case in 2B (x150). (D) TTF-1 was positive in some cells in the same case in 2B (x150). (E) p63 was positive in some cells in the same case in 2C (x150). (F) TTF-1 was positive in some cells in the same case in 2C (x150). (G) ALK was positive in tumor cells as the same case in 2C (x150). (H) ALK was positive in some tumor cells as the same case in 2D (x150).

CK7, Muc5AC, p63 (Fig 4A, 4C and 4E), EGFR, and TTF-1 (Fig 4B, 4D and 4F) were positive in all MEC-like tumors (8/8); p40 was positive in 5 cases (5/8) including 2 scanty positive cases, and ALK was positive in 5 cases (5/8, Fig 4G and 4H); HER-2 was negative in all cases. The Ki-67 labeling index ranged from 3% to 15% (mean, 7.4%) (Table 4).

### 4. FISH findings

**Fig 5:**
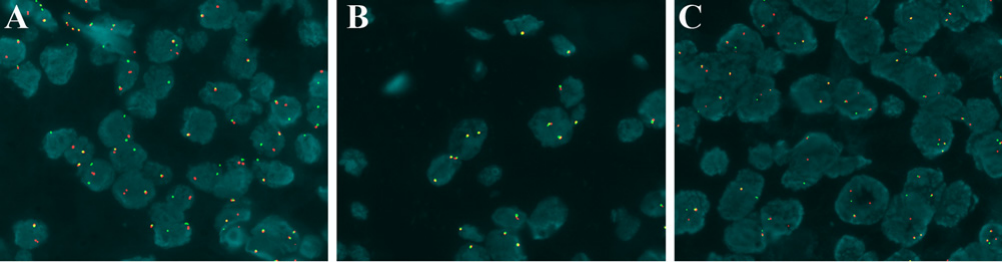
FISH analysis. (A) The MAML2 gene is rearranged in PMEC and shows a disruption of the red and green signals (x1000, oil immersion). (B) The MAML2 gene is not rearranged in MEC-like pulmonary carcinoma and only shows overlapping yellow or green/red fusion signals (x1000, oil immersion). (C) TheALK gene is rearranged in MEC-like pulmonary carcinoma and shows a disruption of the red and green signals (x1000, oil immersion).

Eighteen cases of PMEC had clearly positive FISH signals. MAML2 rearrangement was identified in 12 cases (Fig 5A, 10 low-grade, 2 high-grade) and not identified in the remaining 6 cases (2 low-grade, 4 high-grade). A total of 83.3% of low-grade tumors had MAML2 rearrangement, and 33.3% of high-grade tumors had MAML2 rearrangement. MAML2 rearrangements were not correlated with PMEC grading (P = 0.107, S1 Table).

Six cases of MEC-like pulmonary carcinoma had clearly positive FISH signals, and none had MAML2 rearrangement (Fig 5B). However, ALK rearrangement was identified in all 5 MEC-like ALK-immunostain-positive cases (Fig 5C).

### 5. EGFR, KRAS, BRAF, ALK, PIK3CA, PDGFRA, and DDR2 gene status

No mutations were found within the EGFR, KRAS, BRAF, ALK, PIK3CA, PDGFRA, and DDR2 genes using NGS, Sanger Sequencing, and QPCR in 9 successfully amplified cases.

### 6. Outcomes and survival analysis

Twenty-three cases had follow-up data (Table 3 and S1 Table). Four patients died from the tumor at 7, 30, 33, and 34 months after diagnosis, 2 of which only underwent biopsy and 3 of which developed metastatic PMEC. Among the remaining 19 patients, 1 survived with the tumor for 31 months after diagnosis without surgery or other treatments, and 18 patients were alive without proof of recurrence or metastasis. Three patients were lost to follow-up after biopsy or surgical resection.

**Fig 6:**
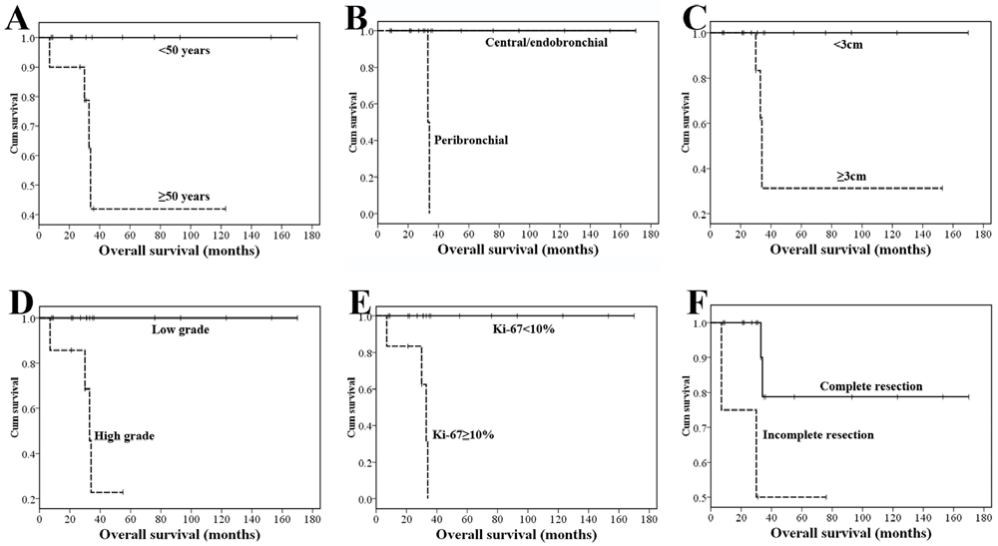
Overall survival of the 23 PMEC patients with follow-up. (A) OS according to age. (B) OS according to growth pattern. (C) OS according to tumor diameter. (D) OS according to tumor grade. (E) OS according to Ki-67 labeling index. (F) OS according to surgical resection.

Both 5-year and 10-year overall survival (OS) were 72.1%. The survival curves are shown in Fig 6A–6F. By univariate analysis, age≥50, peribronchial growth pattern, tumor size≥3 cm, high-grade tumor, and Ki-67 labeling index ≥10% were all adverse prognostic factors in PMEC, while complete resection had a favorable prognostic significance (P<0.05). Gender, lymph node metastasis, MAML2 rearrangement, and chemotherapy (CHT) and/or radiotherapy (RT) had no prognostic significance (Table 6).

**Table 6:** Univariate analysis of overall survival for patients with pulmonary mucoepidermoid carcinoma.

Parameter	Number	OS		
		RR	95% CI	P value
**Age**				**0.022**
≥50	10	1		
<50	13	91.093	0.025–331165.931	
**Gender**				0.873
Male	12	1		
Female	11	0.851	0.117–6.200	
**Tumor growth pattern**				**0.001**
Central/endobronchial	17	1		
Peribronchial	3	7374.899	0.000–3.649	
**Tumor size**				**0.014**
≥3 cm	8	1		
<3 cm	13	0.005	0.000–199.958	
**Lymph node metastasis**				**0.083**
No	18	1		
Yes	1	7.939	0.494–127.593	
**Histological grade**				**0.001**
Low grade	16	1		
High grade	7	288.566	0.009–9442152.996	
**Ki-67 labeling index**				**0.000**
≥10%	6	1		
<10%	17	0.001	0.000–5847.718	
**Surgery**				**0.041**
Complete resection	19	1		
Incomplete resection	4	6.243	0.850–45.848	
**RT/CHT**				0.333
Yes	9	1		
No	14	2.928	0.300–28.553	
**MAML2 rearrangement**				0.213
Yes	11	1		
No	5	51.881	0.001–5166945.856	

RR, relative risk

CI, confidence interval

RT, radiotherapy

CHT, chemotherapy

## Discussion

Primary salivary gland-type tumors of the lung, including MEC, adenoid cystic carcinoma (ACC), and epithelial-myoepithelial carcinoma (EMEC), are rare [1]. They differ from the more common types of lung cancer, such as adenocarcinoma and squamous cell carcinoma, in that the former tend to occur in younger patients, to affect the central airways, and to have a more indolent nature [27]. MEC is the most common salivary gland malignancy. Although it is most commonly identified in the head and neck, it can occur in many sites of the body, including the breasts, lungs, skin, and thymus [28, 29].

As a malignant tumor of bronchial gland original, PMEC was first described in 1952 by Smetana [30]. Clinically, PMEC occurs over a broad age range of 3–78 years with a peak age of diagnosis in the third and fourth decades [1, 31–36]. Although some studies reported a male predominance [31–33] or an equal sex distribution [34–36], most reports failed to demonstrate a clear predilection based on gender. Symptoms primarily include bronchial irritation and obstruction, including cough, wheezing, hemoptysis, and postobstructive pneumonia [1]. In our series, ages ranged from 12–79 years, and there was no gender predilection. Cough, hemoptysis, and dyspnea were the most common manifestations. The tumors were mainly located in the lobe and segmental bronchus, and 85% of patients who underwent pulmonary function tests were normal.

In the 2015 WHO classification, PMEC is divided into low- and high-grade types on the basis of morphology and cytology. In our series, we graded PMEC according to the WHO criteria. Our study showed that low-grade PMEC tumors (69.2%) were more common than high-grade PMEC, and all low-grade tumors had a central/endobronchial growth pattern. All cases contained mucous, epidermoid, and intermediate cells and lacked keratinization, except in one patient where it accompanied dedifferentiation. Foci of calcification or ossification have been reported to be present within the tumor, and the incidence of calcification in PMEC was much higher than in the more common forms of pulmonary carcinoma [1]. In our series, calcification was only detected in low-grade tumors, at 61.1% (11/18) of low-grade tumors. We hypothesized that the phenomenon of calcification might be a predictor of indolent behavior in PMEC. We also found that the accompanying stroma was often hyalinized, which might have an amyloid-like appearance in PMEC. Recently, Yamatani et al. reported 8 cases of pulmonary carcinoma with a MEC-like component, which consisted of solid P63-positive, TTF-1-negative nests with mucin-filled cysts or a cribriform-like structure. They confirmed that the 8 cases were unique adenosquamous carcinomas and clinicopathologically differed from ordinary PMEC [37]. Other studies contain cases that were re-diagnosed as another tumor after being originally identified as PMEC cases [15]. All 8 cases initially diagnosed as PMEC in our pathology files and re-diagnosed as MEC-like pulmonary carcinoma in our study, had mucin-filled cysts or mucin-filled cells in solid nests. Based on strict morphological criteria, 2 cases were adenosquamous carcinoma with a small clearly squamous carcinoma component, and 6 cases were adenocarcinomas containing a variable degree of clearly adenocarcinoma components. Moreover, MEC-like pulmonary carcinoma showed a location preference in the lung, and there were distinct differences between it and PMEC in tumor location.

There were some markers that could help ensure a proper diagnosis. In our series, TTF-1 was negative in all PMEC cases, whereas it was positive in all cases of MEC-like pulmonary carcinoma. Our results confirmed that TTF-1 was very helpful in discriminating PMEC from primary pulmonary adenocarcinoma and adenosquamous carcinoma, including MEC-like carcinoma. p63 is demonstrated to be positive in MEC. However, it is also expressed in primary pulmonarycarcinoma, including squamous carcinoma, adenosquamous carcinoma, and a minor proportion of adenocarcinoma, and may lead to misdiagnosis. Our results showed that p63 was positive in all 26 PMEC cases and all primary MEC-like pulmonary carcinomas, indicating that p63 might have limited value in the differential diagnosis between PMEC and MEC-like pulmonary carcinoma.p40 is another marker used in the diagnosis of PMEC [15]. It is considered to be more specific than p63 for squamous differentiation, and thus help avoid misinterpreting p63-positive adenocarcinoma as squamous cell carcinoma [38]. In our series, all PMEC cases were positive for p40, but 5 MEC-like pulmonary carcinomas were also positive, and the result suggested that p40 might have limited value in the differential diagnosis between PMEC and MEC-like pulmonary carcinoma. Although we could see hyalinization stroma in all lesions, no collagen IV positive material was found in any of the 26 cases, and no calponin-positive myoepithelial cells were found. This could aid in the differential diagnosis with other lung salivary gland-type carcinomas, such as ACC and EMEC. Some research has found that Muc5Ac may mark respiratory-type mucin. It is usually expressed in bronchial epithelium and the mucus-secreting component of bronchus-associated salivary glands [39, 40]. Our results also found that Muc5Ac could help to identify mucous cells in PMEC. HER2 protein overexpression and gene amplification have been reported in 2.6–37.9% and 9.5–20.7% of MEC, respectively [19–21] and might be associated with poor outcome [41]. In our series, HER2 protein was negative in all cases, which needs further clarification in larger samples.

Interestingly, ALK gene rearrangement was revealed by both IHC and FISH in 5 of our 8 MEC-like cases, including 4 adenocarcinomas and 1 adenosquamous carcinoma. This is different from PMEC, in which we found no ALK protein expression, which was the optimal screening tool for detecting ALK rearrangements. So far, there has been only one ALK-rearranged PMEC reported by previous publications [42]. Our study explored the relationship between ALK and PMEC or MEC-like pulmonary carcinoma in a relatively large series, and suggested that ALK rearrangement was more common in MEC-like pulmonary carcinoma than PMEC. In the literature, ALK-rearranged lung cancers make up only 3–7% of all NSCLC cases. However, some studies reported that cribriform structure, prominent extracellular mucus, and any type of mucous cell patterns are sensitive and/or specific for predicting ALK rearrangement. A few ALK-rearranged tumors coexpressed p63 and TTF1 in the adenocarcinoma component [43, 44]. Consistent with these studies, we found in our series that all four ALK-rearranged adenocarcinomas had both mucous cells and p63-positive cells. Because of the therapeutic significance of crizotinib, an ALK tyrosine kinase inhibitor, in ALK rearrangement cases, the differential diagnosis of MEC-like lesions from PMEC and other types of lung adenocarcinoma might be critical. Nevertheless, given the small sample size and the heterogeneous histology, the results should be regarded with caution and validated in a large series.

MAML2 rearrangement is the most common molecular genetic event in MEC, and it is commonly identified in 30%-100% of cases. Some studies have suggested that the MAML2 rearrangement is much more common in low-grade than high-grade MEC and that the presence of a MAML2 rearrangement identifies a biologically distinct group of MEC with a less aggressive clinical behavior [7–9]. In our series, a MAML2 rearrangement was identified in most PMEC cases and exhibited a trend towards being found in low-grade more than high-grade PMEC. However, the result did not reach statistical significance. However, recent studies identified MAML2 rearrangement in high-grade MEC at high levels [10, 11, 15], which might be due to their relatively small sample sizes. FISH analysis revealed no MAML2 gene rearrangement in all 8 MEC-like carcinomas and confirmed that the nature of the MEC-like carcinoma is different from PMEC. Therefore, our study indicated that it was necessary to distinguish PMEC and MEC-like carcinoma by a combination with morphology, immunostains, such as TTF-1, and MAML2 rearrangement.

Genetic alterations associated with the development of NSCLC have been extensively characterized. The driver genes involved include EGFR, KRAS, BRAF, ALK, PIK3CA, DDR2, MEK, and PDGFRA [16, 17]. However, the mutational status of these driver genes in PMEC has not been well characterized. Although EGFR protein overexpression has been reported in 30–78% of MEC cases, most studies from Western populations have found that EGFR mutations are absent in both pulmonary and salivary MEC [22, 24]. Interestingly, 9 PMEC cases with EFGR mutations have been identified in Asian populations [23, 25]. In addition, there have been a few studies demonstratingthat an EGFR copy number gain due to chromosome 7 polysomy was correlated with the histological grade of MEC [20]. In our series, although EGFR protein was overexpressed in 11 PMEC tumors, no mutations in EGFR were detected. Although few studies found alterations of KRAS in MEC [24], there were no KRAS mutations in our series. Genetic alterations of PIK3CA, BRAF, ALK, DDR2, and PDGFRA have been reported to be associated with the development of NSCLC. However, their alterations in MEC have not yet been investigated. In our series, there were also no mutations in PIK3CA, BRAF, ALK, DDR2, and PDGFRA. Our study suggested that EGFR, KRAS, BRAF, ALK, PIK3CA, PDGFRA, and DDR2 might not be the driver genes in PMEC.

Surgery is the preferred treatment, and no evidence has proven the benefits of CHT or RT [1, 28]. Low-grade PMEC has an indolent clinical course, and radical surgery can be curative. The prognosis for patients is much better than with the more common lung cancers. Unfortunately, high-grade PMEC has a worse clinical outcome after surgery. Prognostic factors appearing to predict poor survival include the histological grade, TNM stage, completeness of resection, lymph node metastasis, and age [27, 31–33, 35]. Xi et al. reported 21 cases of PMEC and reported that lymph node metastasis is the most important prognostic factor of PMEC [35]. In our series, both 5- and 10-year OS was 72.1%, and from univariate analysis, age≥50, peribronchial growth pattern, tumor size≥3 cm, high-grade tumor, and Ki-67 labeling index ≥10% were adverse prognostic factors in PMEC. A completeness of resection had a favorable prognostic significance (P<0.05). Inconsistent with previous studies, lymph node metastasis was not a prognostic factor in our series, which might because our cases were found in a relatively early stage and only one case was found with lymph node metastasis.

Our study has some limitations. First, the number of samples in the study was relatively small because of the low incidence. Second, because it was a retrospective study with a span of 15 years, some data were not readily available. For example, extracting DNA from FFPE blocks in some cases for amplification was not successful. Moreover, given that only 8 MEC-like tumors that had been initially diagnosed as MEC were included in our series, we need to further explore the histopathologic features and molecular genetic characteristics of MEC-like tumors in a large sample.

## Conclusions

PMEC is a primary malignant pulmonary tumor with a relatively good prognosis and is clinicopathologically characterized by the bronchial location, the presence of mucous cells, and a lack of keratinization. p63, p40 and Muc5Ac are expressed, and TTF-1 is not expressed, in PMEC. The MAML2 rearrangement is the main genetic event in PMEC, and it tends to be more frequently found in low-grade PMEC than in high-grade PMEC. Although the morphological distinction of PMEC from its mimics can sometimes be challenging, the location preference, immunophenotype, and molecular genetics may be helpful for the differential diagnosis between PMEC and MEC-like pulmonary carcinoma, which is critical for therapeutic and prognostic considerations.

## Supporting Information

S1 Table Clinicopathological, immunohistochemical, and fluorescence in situ hybridization detailed data of 26 patients with pulmonary mucoepidermoid carcinoma. (PDF)

## Abbreviations

ACC: adenoid cystic carcinoma
CRTC1/3: cAMP-response element binding protein-regulated transcriptional co-activator 1/3
DDR2: discoidin domain receptor 2
EGFR: epidermal growth factor receptor
EMEC: epithelial-myoepithelial carcinoma
KRAS: Kirsten rat sarcoma v homolog iral oncogene
MAML2: mammalian mastermind-like protein 2
HER-2: human epidermal growth factor 2
FISH: fluorescence in situ hybridization
MEC: mucoepidermoid carcinoma
NGS: next gerneration sequencing
NSCLC: Non-small cell lung cancer
OS: overall survival
PDGFRA: platelet-derived growth factor receptor alpha
PIK3CA: phosphatidylinositol 3-kinase catalytic alpha polypeptide
PMEC: pulmonary mucoepidermoid carcinoma
QPCR: quantitative polymerase chain reaction
RT: radiotherapy
TTF-1: thyroid transcription factor-1
