# Supplementary material for: Primary Pulmonary Mucoepidermoid Carcinoma: Histopathological and Moleculargenetic Studies of 26 Cases
Source: PLoS One. 2015 Nov 17;10(11):e0143169. doi: 10.1371/journal.pone.0143169 (PMC4648574; doi:10.1371/journal.pone.0143169)
Supplement: S1 Table — (PDF) [file pone.0143169.s001.pdf]

**S1 Table. Clinicopathological, immunohistochemical, and fluorescence in situ hybridization detailed data of 26 patients with pulmonary mucoepidermoid carcinoma**

| No. | Age, yr / gender | Location | Symptoms | Surgery | Histological grade | IHC findings | | | | | | | | | MAML2 arrangement | Follow -up time, mo | RT/ CHT | Recurrenceor metastasis (sites) | Death |
|---|---|---|---|---|---|---|---|---|---|---|---|---|---|---|---|---|---|---|---|
| | | | | | | TTF-1 | CK7 | Muc5Ac | p63 | p40 | EGFR | ALK | HER-2 | Ki-67 | | | | | |
| 1 | 61/ Female | Lower right lobe | Cough, hemoptysis , dyspnea | Yes | High-grade | - | + | + | + | + | + | - | - | 15 | No | 34 | No | Yes (bone, adrenal gland, and lymph node) | Yes |
| 2 | 46/ Female | Left main bronchus | Dyspnea | Yes | High-grade | - | + | + | + | + | + | - | - | 7 | No | 55 | No | No | No |
| 3 | 34/ Male | Trachea | Cough, hemoptysi | Yes, focal resection | Low-grade | - | + | + | + | + | - | - | - | 5 | Yes | 76 | RT | No | No |
| 4 | 51/ Female | Trachea | Dyspnea | Yes | Low-grade | - | + | + | + | + | + | - | - | 3 | No | 36 | No | No | No |
| 5 | 79/ Male | Lower left segmental bronchus | Cough, hemoptysi | Yes | High-grade | - | + | + | + | + | + | - | - | 25 | No | 33 | No | Yes (brain) | Yes |
| 6 | 45/ Female | Middle right segmental bronchus | Cough, fever | Yes | Low-grade | - | + | + | + | + | - | - | - | 2 | Yes | 93 | RT | No | No |

| | | | | | | | | | | | | | | | | | | | |
|---|---|---|---|---|---|---|---|---|---|---|---|---|---|---|---|---|---|---|---|
| 7 | 39/ Female | Lower left segmental bronchus | Cough | Yes | Low-grade | - | + | + | + | + | - | - | - | 5 | Unknown | 153 | No | No | No |
| 8 | 58/ Male | Upper right lobe | Chest pain | Yes | High-grade | - | + | + | + | + | - | - | - | 80 | No | 0 | Unknown | Unknown | Unknown |
| 9 | 40/ Male | Middle right segmental bronchus | Hemoptysis | Yes | Low-grade | - | + | + | + | + | - | - | - | 5 | Yes | 35 | CHT | No | No |
| 10 | 17/ Female | Trachea | Hemoptysis, dyspnea | Yes | Low-grade | - | + | + | + | + | + | - | - | 5 | Unknown | 21 | No | No | No |
| 11 | 42/ Male | Upper right segmental bronchus | Cough, hemoptysis, dyspnea | Yes | Low-grade | - | + | + | + | + | - | - | - | 5 | Unknown | 8 | No | No | No |
| 12 | 32/ Female | Lower left lobe | Cough, hemoptysis | Yes | Low-grade | - | + | + | + | + | - | - | - | 2 | Yes | 22 | No | No | No |
| 13 | 50/ Female | Upper left segmental bronchus | Cough, hoarseness | No, only biopsy | High -grade | - | + | + | + | + | + | - | - | 12 | Unknown | 30 | CHT | Yes (bone) | Yes |
| 14 | 27/ Male | Upper right lobe bronchus | Asymptom | Yes | Low-grade | - | + | + | + | + | - | - | - | 4 | Unknown | 31 | No | No | No |
| 15 | 54/ Female | Lower right lobe | Asymptom | Yes | Low-grade | - | + | + | + | + | - | - | - | 2 | Unknown | 123 | RT/ CHT | No | No |

| | | | | | | | | | | | | | | | | | | | |
|---|---|---|---|---|---|---|---|---|---|---|---|---|---|---|---|---|---|---|---|
| | | bronchus | | | | | | | | | | | | | | | | | |
| 16 | 58/ Male | Lower left lobe bronchus | Cough, hemoptysis, dyspnea, chest pain | Yes | Low-grade | - | + | + | + | + | - | - | - | 3 | Yes | 33 | CHT | No | No |
| 17 | 41/ Male | Lower left segmental bronchus | Cough | Yes | Low-grade | - | + | + | + | + | - | - | - | 2 | No | 170 | CHT | No | No |
| 18 | 12/ Female | Upper left lobe bronchus | Cough | Yes | Low-grade | - | + | + | + | + | - | - | - | 7 | Yes | 0 | Unknown | Unknown | Unknown |
| 19 | 67/ Male | Lower left lobe bronchus | Asymptom | No, only biopsy | High -grade | - | + | + | + | + | + | - | - | 15 | Unknown | 7 | No | No | Yes |
| 20 | 28/ Male | Middle right segmental bronchus | Hemoptysis | Yes | High -grade | - | + | + | + | + | - | - | - | 10 | Yes | 21 | No | No | No |
| 21 | 68/ Female | Trachea | Hemoptysis | Yes | Low-grade | - | + | + | + | + | - | - | - | 5 | Yes | 27 | RT | No | No |
| 22 | 40/ Female | Lower right lobe bronchus | Cough | Yes | Low-grade | - | + | + | + | + | - | - | - | 2 | Yes | 0 | Unknown | Unknown | Unknown |
| 23 | 67/ Male | Lower right lobe | Cough | Yes | High -grade | - | + | + | + | + | + | - | - | 15 | Yes | 30 | No | No | No |

| | | | | | | | | | | | | | | | | | | | |
|---|---|---|---|---|---|---|---|---|---|---|---|---|---|---|---|---|---|---|---|
| 24 | 72/ Male | Lower left lobe bronchus | Asymptom | No, only biopsy | Low-grade | - | + | + | + | + | + | - | - | 5 | Unkown | 31 | No | Uncertain | No (survival with tumor) |
| 25 | 40/ Male | Right main bronchus | Cough, hemoptysis | Yes | Low-grade | - | + | + | + | + | + | - | - | 5 | Yes | 9 | RT/ CHT | No | No |
| 26 | 40/ Female | Lower left lobe bronchus | Cough, dyspnea | Yes | Low-grade | - | + | + | + | + | + | - | - | 7 | Yes | 9 | No | No | No |

RT, radiotherapy

CHT, chemotherapy
